# Evaluating anthropogenic threats to endangered killer whales to inform effective recovery plans

**DOI:** 10.1038/s41598-017-14471-0

**Published:** 2017-10-26

**Authors:** Robert C. Lacy, Rob Williams, Erin Ashe, Kenneth C. Balcomb III, Lauren J. N. Brent, Christopher W. Clark, Darren P. Croft, Deborah A. Giles, Misty MacDuffee, Paul C. Paquet

**Affiliations:** 1 Chicago Zoological Society, Brookfield, IL 60513 USA; 2 Oceans Initiative, Seattle, WA 98102 USA; 3 Center for Whale Research, Friday Harbor, WA 98250 USA; 4 College of Life & Environmental Sciences, University of Exeter, Exeter, Devon, EX4 4QG UK; 5 Cornell Lab of Ornithology, Cornell University, Ithaca, NY 14850 USA; 6 Raincoast Conservation Foundation, Sidney, BC V8L 3Y3 Canada; 7 University of Victoria, Victoria, BC V8P 5C2 Canada

## Abstract

Understanding cumulative effects of multiple threats is key to guiding effective management to conserve endangered species. The critically endangered, Southern Resident killer whale population of the northeastern Pacific Ocean provides a data-rich case to explore anthropogenic threats on population viability. Primary threats include: limitation of preferred prey, Chinook salmon; anthropogenic noise and disturbance, which reduce foraging efficiency; and high levels of stored contaminants, including PCBs. We constructed a population viability analysis to explore possible demographic trajectories and the relative importance of anthropogenic stressors. The population is fragile, with no growth projected under current conditions, and decline expected if new or increased threats are imposed. Improvements in fecundity and calf survival are needed to reach a conservation objective of 2.3% annual population growth. Prey limitation is the most important factor affecting population growth. However, to meet recovery targets through prey management alone, Chinook abundance would have to be sustained near the highest levels since the 1970s. The most optimistic mitigation of noise and contaminants would make the difference between a declining and increasing population, but would be insufficient to reach recovery targets. Reducing acoustic disturbance by 50% combined with increasing Chinook by 15% would allow the population to reach 2.3% growth.

## Introduction

Conservation science is tasked with quantifying the relative importance of multiple anthropogenic threats to species, both to determine if cumulative impacts exceed sustainable levels and to guide effective recovery plans^1–4^. However, cumulative human impacts are often poorly understood and inadequately addressed in conservation and management^5^. Fundamental research is still needed to integrate information on qualitatively different stressors into comprehensive models that reveal the cumulative impacts on measures of population growth, stability, and resilience^6^. Such work is needed, in part, because threats vary widely in their amenity to mitigation. When regulators require users to forego economic opportunities, it is important to have confidence that management actions will achieve the desired effect^7^. One way to accomplish this is to conduct “population viability analyses” (PVA) that use models of population dynamics to evaluate the relative importance of multiple anthropogenic stressors, singly and in combination, so that conservation can be directed toward efforts most likely to promote species recovery^8^. PVA can be a powerful tool for informing management and conservation decisions. However, the detailed population models used in PVA depend on: availability of estimates for demographic rates (both fecundity and survival and the variability in such rates); confidence that observed past rates are predictors of ongoing demography, or that trends can be foreseen; data for quantifying effects of threats on demographic rates; and a population model that adequately captures the key demographic, social, genetic, and environmental processes that drive the dynamics of the population of concern. Nevertheless, even when data on certain aspects of the population or its threats are not available, we can use PVA models to explore possible outcomes across a plausible range of values, and thereby identify which factors might be important and the target of additional research.

The Southern Resident killer whale (*Orcinus orca*, SRKW) population in the northeastern Pacific Ocean is one of the most critically endangered populations of marine mammals in the USA^9^ and Canada^10^. The USA and Canada have listed this transboundary population as Endangered, citing three primary risk factors: lack of the whales’ preferred prey, Chinook salmon (*Oncorhynchus tshawytscha*); chronic and acute underwater noise and physical disturbance (e.g., from ferries, commercial ships, whale-watching boats, fishing boats, and recreational traffic); and high levels of contaminants, including polychlorinated biphenyls (PCBs)^10,11^. A recent Status Review^12^ highlighted also the potential risk to this small, localized population from catastrophic events such as an oil spill. Governments and non-governmental organizations are currently seeking effective conservation measures for this high-profile population. Fortunately, the biological and environmental data available for SRKWs are rich by the standards of any marine mammal population. Long-term annual censuses, with continuous monitoring since 1976, coupled with the specialized diet, have allowed inference of quantitative relationships between prey and various metrics of fecundity and survival^13,14^. Thus, the prerequisites for a robust PVA suitable for guiding conservation are met.

PVA uses demographic models to assess risk to wildlife populations and evaluate the likely efficacy of protection measures, recovery targets, and restoration options^15,16^. We used the Vortex PVA model to examine the dynamics of SRKWs. Vortex^17–19^ is a flexible, individual-based simulation that is freely available. Vortex has been used to set recovery goals and guide actions for many threatened species, including the Mexican wolf (*Canis lupus baileyi*)^20^, Florida panther (*Puma concolor coryi*)^21^, and Florida manatee (*Trichechus manatus latirostris*)^22^. Several recent PVAs on the SRKWs have shown how variability in demography^23^ or inter annual variability in Chinook salmon abundance^12,24,25^ could affect the population. We extend those approaches to consider also the sub-lethal effects of contaminants and acoustic disturbance, and the cumulative impacts of threats and interactions among them.

We first parameterized a Baseline model with demographic rates observed over 1976 through 2014, and tested the sensitivity of population growth to each demographic parameter. We then constructed one model that quantifies the population consequences of all three anthropogenic threats to SRKWs identified in Canadian^10^ and USA^11^ recovery plans. We compared the relative importance of each threat by projecting the population growth across the possible range of each threat. Finally, we used the PVA to explore the degree to which threats would have to be mitigated, alone or in combination, to reach a quantitative USA recovery target of sustained 2.3% growth over 28 years^11^.

## Results

**Figure 1:**
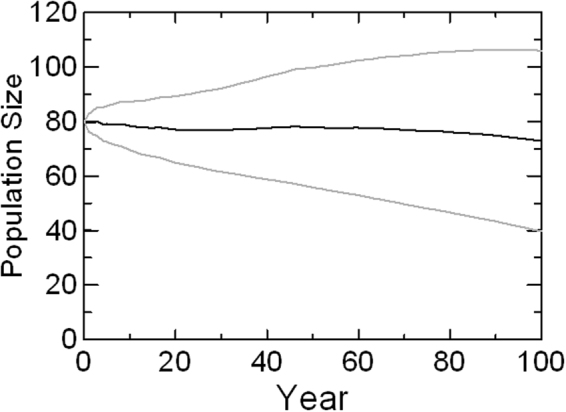
The distribution of 10,000 simulated trajectories with means and SD of the population size for northeastern Pacific Ocean SRKWs projected for 100 years, based on demographic rates observed from 1976 through 2014, applied to a starting population as it existed in 2015.

Five sets of population models and the scenarios examined in each are listed in Table 1. The Baseline model projects mean population growth over the next 100 years of r = −0.002, with variation across years of SD = 0.045 (Fig. 1). These projections match very closely to the rate of r = 0.002, with SD = 0.042, observed over 1976 to 2014. The marginally lower growth in the model can be accounted for by future accumulation of low levels of inbreeding. After 100 years, the projected mean inbreeding coefficient is 0.067, about the same as results from mating between first-cousins. When inbreeding depression was eliminated from the Baseline model, the projected growth was r = 0.002, with SD = 0.043 – nearly identical growth and variation in growth to the trend in recent decades, and thereby confirming that the model replicates accurately the recent dynamics of the population.

**Table 1:** Models of viability of the SRKW population for assessing current viability, sensitivity to anthropogenic threats, and responses to management.

Set	Scenario	Parameters varied	Population growth (r)
Baseline	Baseline	Rates as observed 1976–2015	−0.002
	Sensitivity Tests	See Supplementary Information (S.I.)	See S.I.
Individual Threats	Current	Chinook = 1.0; Noise = 85%; PCB = 2 ppm/y	−0.001
	Chinook	0.6 to 1.3 × baseline	−0.038 to +0.025
	Noise	0 to 100% of time	+0.017 to −0.004
	PCB	0 to 5 ppm/y	+0.003 to −0.008
Cumulative Threats	No Anthropogenic Threats	baseline Chinook; no noise, no PCB; no oil spills; no ship strikes	+0.019
	Low Development	25% decline in Chinook; 92.5% noise; low frequency oil spills and ship strikes (see Table 2)	−0.008
	High Development	50% decline in Chinook; 100% noise; higher frequency oil spills and ship strikes (see Table 2)	−0.017
Demographic Management	Fecundity	1 to 1.5 × baseline	+ 0.016
	Adult Mortality	1 to 0.5 × baseline	+ 0.009
	Calf Mortality	1 to 0.5 × baseline	+ 0.004
Threat Management	Chinook	1 to 1.3 × baseline	+ 0.025
	Noise	85% to 0%	+ 0.017
	PCB	2 to 0 ppm/y	+ 0.004
	Chinook & Noise	1 to 1.3 × Chinook; 42.5% Noise	+ 0.036

Population growth rates are mean r for Baseline, ranges for tests of Individual Threats, means for Cumulative Threat scenarios, and maxima for ranges tested in Demographic Management and Threat Management scenarios.

Sensitivity tests of the influence of each demographic rate in the baseline PVA (Supplementary Information) show that, across the ranges of values tested, variation in fecundity (defined for the model as the mean proportion of adult females giving birth per year) accounts for most (77%) of the uncertainty in population growth rate. Annual adult mortality has some influence on the population trajectories (6%), but because mortality is already close to 0, there is comparatively less opportunity to improve the value of this parameter. Calf (first year) and juvenile (1 y to 10 y) mortality each accounted for about 3% of variation in population growth. Individual variation in reproductive success and temporal fluctuations (EV) in demographic rates had almost no effect on long-term population growth, as would be expected for a very long-lived species in which short-term fluctuations average out over time. Therefore, although our estimates of annual variation in rates are uncertain, refining the estimates would not change any conclusions about the effects of threats on the viability of the population. Given the small population size, inbreeding depression might cause sufficient adverse impact on population viability (6% of the total variance explained) such that it should not be ignored in assessments of long-term population viability. The impact of inbreeding was exacerbated slightly when we did not include avoidance of very close inbreeding (Supplemental Information).

### Individual Threats

**Figure 2:**
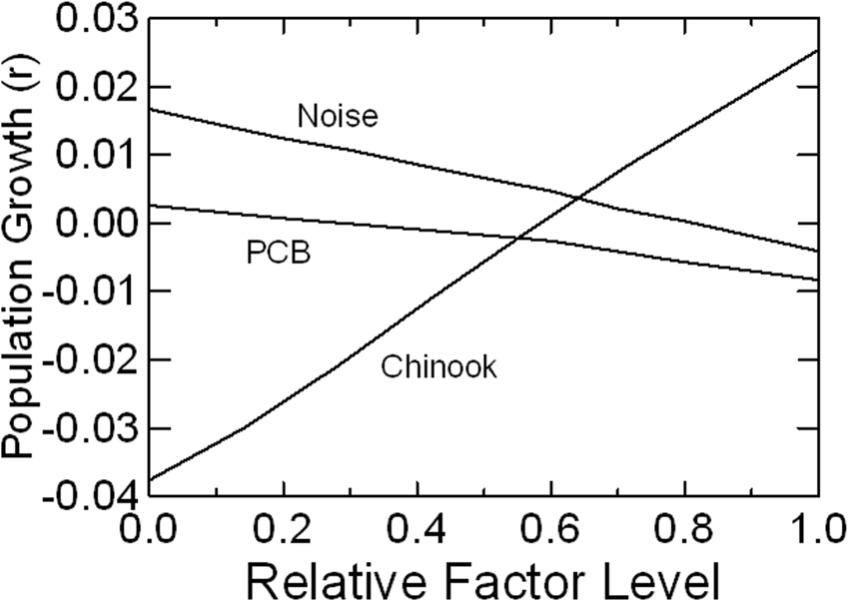
Effect of Chinook prey abundance (index varied from 0.60 to 1.30), noise and disturbance (boats present from 0% to 100% of time), and PCB contaminants (accumulation rate from 0 to 5 ppm/y) on mean population growth, while holding the other two factors at their baseline levels (1.0 prey index, 85% noise, and 2 ppm/y PCB accumulation). The x-axis is standardized to the range tested for each variable.

The set of models that includes estimates for the threats identified in the recovery plans – Chinook prey availability, noise and disturbance, and contaminants – was calibrated so that in the Current Threats scenario the demographic rates at existing threat levels reflect the mean demographic rates observed from 1976 through 2014. Thus, the Current Threats scenario mirrored the simpler Baseline scenario, except that rounding error in estimating effects of threats led to very slight deviation from the Baseline. The levels of these threats were then varied across broad ranges of values to determine which threat would have the greatest impact on population growth. Over the ranges tested, the effects of Chinook prey abundance on fecundity and survival had a greater effect on the population growth rate than did the other two factors (Fig. 2). Noise disturbance acts through decreased feeding efficiency in our model, but has a lesser effect than prey abundance because the maximum impact of boat noise 100% of the time would be to reduce foraging by about 20%. PCB accumulation rates that we tested result in mean levels in adult females of 0 to 132 ppm. Across this range, calf mortality is predicted to rise from about 7% to 50% (see Methods), and this impact shifts population growth from slightly positive to negative.

### Cumulative Threats

**Figure 3:**
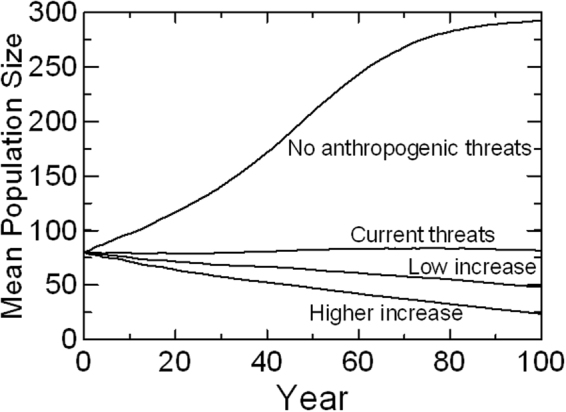
Mean projected SRKW population sizes for scenarios with (from top to bottom): no anthropogenic noise or contaminants; current Chinook abundance, noise, and PCBs; reduced Chinook, increased noise, and additional threats of oil spills and ship strikes as estimated for low level impacts of future industrial development; and these increased and additional threats with higher level impacts of development.

Threats may interact, such that cumulative effects differ from those projected based on the summation of individual impacts. Full exploration of all of the possible interactions among the threats to the SRKW is not warranted at this time because individual threats are not yet well quantified. As more data on the above threats and other threats are acquired, management authorities can use the PVA framework to examine specific interactions of interest or full statistical analysis of all possible interactions^26^. To illustrate how cumulative threats can be assessed within the PVA model, we examined combinations of threat levels that represent the cumulative impacts of multiple threats for a few sample scenarios. We compared the Current Threats to a scenario with no anthropogenic threats and to scenarios with an increase in current threats and the addition of new threats. Figure 3 compares the population trajectory for the Current Threats with a scenario in which noise and PCB contamination were set to 0, and with two scenarios that describe levels of threat that could occur with proposed further industrial development and climate change. Table 2 shows the mean growth rates, probabilities of decline below 30 animals, and probabilities of extinction within 100 years under these scenarios.

**Table 2:** Measures of viability of the SRKW population over 100 years under scenarios of minimal anthropogenic threats, current threats, and two levels of increased threats due to development.

	Threats modelled					Population projection		
Scenario	Chinook trend	Noise	PCB (ppm/y)	Oil spill (big; small)	Ship strikes	Population growth (r)	Probability extinct	Probability final N < 30
No anthropogenic threats	constant	0	0	0	0	0.019	0	0
Current threats	constant	85%	2	0	0	−0.001	0	5%
Low increase	−25% in 100 y	92.5%	2	0.21%; 1.08%	1 per 10 y	−0.008	5%	31%
Higher increase	−50% in 100 y	100%	2	0.42%; 2.16%	2 per 10 y	−0.017	25%	70%

See text for explanation of threats modelled.

The population could show robust growth if all anthropogenic threats were removed, but has no growth under current threat levels (Fig. 3). The combination of increased and additional threats expected under planned further industrial development in the habitat of the SRKW would cause population decline.

### Demographic Management

**Figure 4:**
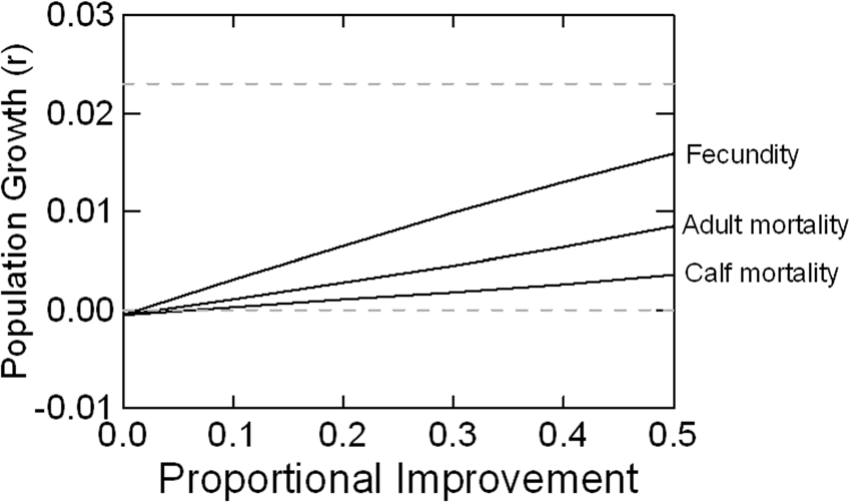
Mean population growth for SRKW achieved by improvements in demographic rates. Fecundity was increased from baseline to 1.5x baseline; mortality rates were decreased from baseline to 0.5x baseline. Dashed lines indicate a stated recovery target (2.3% growth) and r = 0.

The potential benefits of improvements in the primary demographic rates were examined in a set of Demographic Management scenarios. The demographic analyses indicate that reaching the SRKW recovery target of 2.3% growth is impossible by improving any single rate by a plausible amount, although increased fecundity would have the greatest positive influence on population growth (Fig. 4). To reach the recovery target, sustained mitigation of threats will be necessary to promote both increased fecundity and reduced mortality.

### Threat Management

**Figure 5:**
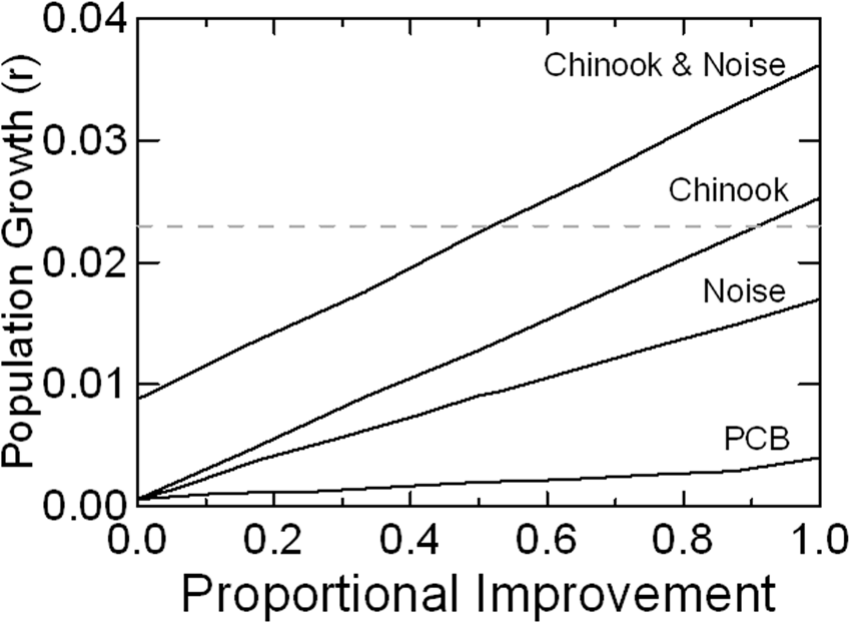
Mean population growth for SRKW achieved by mitigation of anthropogenic threats. Threat reductions are scaled on the x-axis from no reduction to the maximum reductions tested: Chinook abundance increased up to 1.3x the long-term mean; noise disturbance during feeding was reduced from 85% to 0; and PCBs were reduced from accumulation rates of 2 ppm/y to 0. The top line shows growth rates under a combination of varying levels of improved Chinook abundance plus mitigation of noise to half the current level.

Improvements in demographic rates would need to be achieved by management actions that reduce threats or otherwise enhance the environment for SRKW. We therefore examined how population growth would respond to reductions in the levels of current threats. To achieve the recovery goal by increasing Chinook abundance alone would require a return to nearly the highest rates of Chinook abundance observed since 1979 (Fig. 5). If eliminating acoustic disturbance while maintaining current levels of Chinook abundance were possible, annual population growth could reach 1.7%. Removal of PCBs from the habitat would result in marginally positive (0.3%) growth, but the effect is much smaller than the impact of reduced noise and disturbance or increased Chinook abundance. Complete removal of both acoustic disturbance and PCBs is predicted to result in 1.9% growth. Therefore, reaching the recovery target without increasing Chinook salmon numbers is likely impossible. Reducing acoustic disturbance by 50% and simultaneously increasing Chinook by more than 1.15x would allow the population to reach the 2.3% growth target. Other combinations of mitigation should be explored by management authorities as conservation options are identified.

## Discussion

The SRKW population has experienced almost no population growth during the past four decades, and it declined in the last two decades. Intensive monitoring of the population since 1976 provides the information for construction of a detailed PVA model that closely replicates the observed population dynamics, and thereby provides a basis for projections under scenarios of increased anthropogenic threats or, conversely, increased mitigation actions. Models projecting population changes based on average demographic rates and fluctuations in those rates project that under the *status quo* the population will most likely remain near its current size. However, our use of baseline demographic rates averaged across 38 years of monitoring might give an overly optimistic projection for the SRKW if rates have deteriorated in recent years. A population projection based on demographic rates observed through 2011 projected a 1% annual mean growth^25^, but a recent Status Review^12^ projects a decline of 0.65% per year if demographic rates (such as recently lower fecundity) remain as they have been during 2011–2016. If ongoing monitoring indicates that these are not just short-term fluctuations in rates, then assessments of current viability, vulnerability to new or increased threats, and measures needed to achieve recovery will need to be revised.

When examined over ranges that encompass plausible improvements, the demographic parameter that presents the better opportunity for a large benefit to population growth is fecundity, rather than mortality. This finding is similar to a study of two bottlenose dolphin (*Tursiops aduncus*) populations off Australia, which found that variability in reproduction was more important than variability in mortality in driving differences between the populations^27^. There is simply more potential for improving reproduction than for improving adult survival when survival is already close to 1. Even complete elimination of adult mortality in the SRKW (not a biological possibility) would result in a population growth rate of 1.8%, still below the recovery goal of 2.3% growth. Although recovery cannot be achieved solely by improving adult survival, any decline in adult survival caused by new or exacerbated threats could have serious consequences for the population.

The PVA was useful for exploring scenarios representing the three main anthropogenic threats – prey limitation, acoustic and physical disturbance, and PCBs – that might worsen with increased development, or could be mitigated through management. Across the ranges of threat levels that we examined, reduction of the prey base was the single factor projected to have the largest effect on depressing population size and possibly leading to extinction, although either higher levels of noise and disturbance or higher levels of PCB contamination are sufficient to push the population from slow positive growth into decline. If additional threats from proposed and approved shipping developments (such as catastrophic and chronic oil spills, ship strikes, and increased vessel noise) combine with the predicted decline of Chinook due to climate change^28^, then the population could decline by as much as 1.7% annually, have a 70% probability of declining to fewer than 30 animals, and have a 25% chance of complete extirpation within 100 years.

Mitigating multiple anthropogenic threats sufficiently to reach the recovery target will be difficult. The PVA is a useful way for managers to identify priorities for future research, and to focus conversations with ocean users and other special interests about the most pragmatic ways to promote recovery of endangered species. Those discussions must be integrated with considerations of feasibility, cost, societal impact, and timeframe for effective implementation. If a threat cannot be mitigated in a timescale relevant to conservation, or if costs are so high that they are prohibitive, thinking of those intractable problems as “fixed costs” in a cumulative impact management framework^4^ might be useful. For example, our model results show that eliminating PCBs would provide less benefit to SRKWs than improving salmon returns or reducing anthropogenic noise and disturbance. This is fortuitous because imagining a way to eliminate PCBs that are persistent in the ecosystem is problematic^29^, even though levels in tissues of SRKWs have been slowly declining in recent decades^30^. Identifying fixed costs that are difficult or impossible to mitigate allows a practical discussion about how to rank recovery actions among the anthropogenic factors that can be managed.

Of the three threats we considered, across wide but plausible ranges of each, salmon abundance is the greatest factor affecting SRKW population dynamics. Previously reported correlations of demographic rates with Chinook abundance^13,14,24^ were used to parameterize our model, and Wasser *et al*.^31^ recently offered insights into a mechanism that could cause the effect on fecundity: hormone levels indicate that SRKWs experience nutritional stress related to periods of lower abundance of Chinook prey and that this stress results in fewer successful pregnancies. Our PVA model estimated that SRKW recovery cannot be achieved without reaching the highest levels of salmon abundance observed since 1979, which was 30% higher Chinook salmon abundance than the long-term average between 1979 and 2008. This model result allows managers to focus discussions on whether achieving such a high sustained level of salmon abundance is attainable, and if so, how to achieve it. For example, removal of a hydroelectric dam on the Elwha River in the state of Washington is expected to increase spawning habitat for all five wild Pacific salmon species in the Salish Sea, but discussions about dam removal began in the 1960s^32^ and the cost was in the hundreds of millions of US dollars. Restoration of spawning and rearing habitat could improve growth and survival of wild, juvenile salmon, but this takes political will, time, and money^33^. Improvement of marine survival of juvenile salmon might be possible by better management of net-pen salmon aquaculture sites that host and amplify viruses and parasites that have the potential to reduce survival of wild salmon^34,35^. Reducing Chinook harvest could provide an interim and strategic opportunity to rebuild depressed wild Chinook salmon runs and increase the number of Chinook available to whales in terminal areas like the Salish Sea^36^. Harvest reductions without longer term rebuilding plans might be an incomplete measure in places where Chinook harvests are already low due to abundance concerns or other constraints^37^.

The SRKW population could be adversely affected by any new threats and further intensified impacts of the anthropogenic threats that we did assess. For example, pollutants other than PCBs might affect the population, and PCBs are known to have adverse effects beyond just reduced infant survival – such as reduced immune function^38^. However, other than calf survival, sufficient data are not yet available on the impacts of PCBs on demographic rates to allow incorporation of those threats in the population model. Moreover, threats to the population likely interact, perhaps in non-linear ways. For example, cetaceans that are food-limited might mobilize more lipids, and this will change the accumulated loads and harmful effects of PCBs and other organic pollutants. Similarly, reduction in foraging success because of boat noise might be of little consequence if prey is abundant, but could be critical if killer whales have difficulty procuring enough prey. If we can obtain data on additional threats and the interactions among threats, such effects could be included in the PVA models. At present, given that only estimates of approximate average effects of some threats are included in the model, inclusion of higher level interactions is premature.

While acknowledging that we examined only the identified primary threats to the SRKWs and that we cannot yet fully assess possible complex interactions among those threats, an important finding from our PVA is that reaching the recovery target will likely require mitigation of multiple threats. For example, the PVA projects that a 50% noise reduction plus a 15% increase in Chinook would allow the population to reach the 2.3% growth target. Noise is a particularly attractive issue to address in a management context, because it is amenable to several possible mitigation scenarios^39,40^. With respect to noise from commercial shipping, preliminary calculations suggest that the distribution of source levels of individual ships follows a power law, implying that quieting the noisiest ships will reduce overall noise levels by a disproportionate amount^41^. Identifying the noisiest ships operating in SRKW critical habitat^42^ and creating incentives to reduce their noise outputs through speed restrictions and maintenance might generate considerable reductions in noise levels. The International Maritime Organization and the International Whaling Commission have urged nations to reduce the contribution of shipping to ocean ambient noise, with some countries adopting a pledge to reduce anthropogenic noise levels by 50% in the next decade^43^. However, from the perspective of a foraging killer whale that emits high-frequency (18-32 kHz) echolocation clicks to detect and capture salmon, high-frequency noise from small, outboard vessels that follow whales might cause a greater reduction in a killer whale’s foraging success than low-frequency (<1 kHz) background noise from commercial shipping^44^.

Clearly, even without new or increased external threats, the SRKW population has no scope to withstand additional pressures. The current situation for SRKWs gives little cause for optimism. This is likely to worsen, given the energy-related project proposals already approved for the region^45^, which will increase broadband ocean noise levels and the risk of ship strikes and oil spills^46^. Our models of the additional threats expected with a proposed increase in oil shipping show that these threats will push a fragile population into steady decline. Obviously, countering such additional threats sufficiently to achieve SRKW population recovery would require even more aggressive mitigation actions than if there were no such increasing threats to the population.

The case study we present offers an unusual opportunity to examine multiple anthropogenic threats in a wildlife population that is extremely data-rich by the standard of any marine ecology study^5^. One threat (the impact of prey abundance through the prey-demography link) has been well studied for decades. Another (acoustic disturbance) is relatively well appreciated in that there are documented relationships between higher noise level and reduction in foraging success. However, a conceptual step is required to convert the reduction in foraging to a reduction in prey acquisition. Full consideration of noise impacts would need to include complex interactions among reduced foraging time, reduced detection space, and reductions in prey availability. The third kind of threat (population consequences of PCBs and other persistent pollutants) relies on very few data points to calibrate the effect of the PCBs only on whale calf survival, which underestimates the total population consequences of contaminants in two ways. Lack of concentration-response studies on compounds other than PCBs hinder our ability to model population consequences of PBDEs or other contaminants. Similarly, existing studies do not allow us to predict effects of contaminants on pregnancy rate or adult mortality. This spectrum of data-rich to data-poor steps in predicting population consequences of multiple stressors is ubiquitous in conservation and ecological studies^2,47^. The funding to fill knowledge gaps with empirical data may be lacking, or in the case of critically endangered species, time to wait for science to fill data gaps may be insufficient^48^. Some authors use expert elicitation^49,50^ to fill data gaps. Expert opinion or examination of hypothetical, but plausible scenarios should be used to augment rather than replace the available data.

The case study presented here illustrates the use of PVA as a method to inform difficult conservation decisions, by simulating across plausible ranges of uncertainty. For example, sensitivity analyses revealed that some factors (e.g., individual variability in breeding success) have no effect, and such knowledge gaps should not be a barrier to management action. Given our inability to manage some insidious threats, such as persistent organic pollutants that are already in the environment, it is reassuring that the model predicts that this stressor has the smallest adverse impact on the population, at least via the pathway of reduced calf survival. The PVA can focus priority research on questions that make a practical difference. Studies of foraging efficiency under varying levels of anthropogenic disturbance are needed only because the population is prey-limited. If doubling Chinook salmon numbers were possible, and returning them to levels seen in the 1920s^51^, consideration of other anthropogenic impacts on the whales’ foraging efficiency might not be necessary. Alas, this is not a realistic scenario, and the model therefore points to the importance of including both improvement in prey abundance and reduction in noise as the more effective mitigation pathway.

Unfortunately, focus on only the immediate, tractable threats is all too common in conservation. For example, conservation of grizzly bears (*Ursus arctos horribilis*) in the continental United States focuses on roads and development activities, but the primary concern is that the species has been absent from most of its range since the 1800s^52^. Similarly, the current small size of the SRKW population was not caused by lack of salmon. The whales’ depleted status is due in large part to the legacy of an unsustainable live-capture fishery for display in aquariums^53^. Salmon, noise, and contaminants are important factors that can prevent recovery. Many policies, including the US National Environmental Policy Act, require regulators to consider the effect of a proposed activity “which results from the incremental impact of the action when added to other past, present, and reasonably foreseeable future actions regardless of what agency (Federal or non-Federal) or person undertakes such other actions (40 CFR § 1508.7).” Allocating impacts among multiple ocean user sectors may be difficult, but in the case study we present, the population is sufficiently imperilled that it has little or no scope for tolerating additional stressors.

## Methods

The SRKW population is closed to immigration and emigration, every individual in the population is known, and the population has been censused annually for decades^11^. Individuals were identified by their unique fin shapes, saddle patches, and the presence of any nicks or scratches, and sexed using distinctive pigmentation patterns around the genital slits. Male and female offspring remain within the natal, matrilineal unit, although mating occurs within and between these pods. The term “resident” refers to their residency in inshore waters of southern British Columbia (Canada) and Washington state (USA) in the summer months, when they feed almost exclusively on Chinook salmon^13,14,54,55^. Given that there is no dispersal from the population^56^, mortality was recorded if an individual’s matriline was observed in the population within a year but the individual did not appear.

We used values of demographic parameters calculated from the census data to build the population model in the Vortex PVA program^17,18^. We included temporal variation in demographic rates (“environmental variation”), based on inter-annual variability in parameters observed since 1976, and we included individual variation in age of maturity and probability of reproductive success. The Vortex simulation model of possible future population trajectories includes demographic stochasticity (binomial variation in individual fates); random assignment of sex and a bi-sexual mating system, resulting in fluctuations in sex ratio and mate availability that can affect small populations; and projections of loss of genetic diversity, allowing for inclusion of inbreeding depression. We quantified population growth as the mean exponential rate of increase (r = ln[N_t+1_/N_t_]).

Modelling was conducted in stages. First, a “Baseline” model was developed to represent the population trajectories if demographic rates remain the same as have been observed in recent decades. We confirmed this Baseline model by comparing simulated dynamics with recent population trends. Secondly, we conducted sensitivity tests on uncertain demographic rates in the model to determine which parameters had large effects on the projected population growth. Thirdly, we used a set of models of Individual Threats that tested ranges of values for the primary threats identified in the recovery plans to determine which would have the greatest effects on population projections. Fourthly, we examined Cumulative Threats scenarios to project the fate of the population if further industrial development increases existing threats and adds new ones. A set of Demographic Management scenarios was then examined to determine the population growth that could be achieved by improvements in demographic rates. Finally, we explored Threat Management scenarios to assess the plausibility of reaching sustained annual population growth of 2.3% given various options for increasing salmon abundance, reducing ocean noise levels, or reducing contaminant levels. The following section describes key parameter estimates used in the model. More detailed description of the modelling methods is presented in Supplementary Information. The input files for the Vortex project are available at http://www.vortex10.org/SRKW.zip and from the Dryad Digital Repository at https://doi.org/10.5061/dryad.46vq7.

### Baseline PVA

We started the simulations with the ages, sexes, and pod membership of the killer whales living in 2015. We specified the mother of each animal, where known (for 76 of 80 living animals)^57^. Based on previous genetic data on paternity^58^, we specified in the simulation that females would not mate with their father, a son, or a maternal half-sibling. What effect lower levels of inbreeding or the inevitable accumulated inbreeding in a closed population will have on any cetacean is unknown. We modelled inbreeding depression as being caused by recessive lethal alleles, with 6.29 “lethal equivalents” (the negative of the slope of log(recruitment) against the inbreeding coefficient), the mean combined effect of inbreeding on fecundity and first-year survival in a survey of impacts on wild species^59^.

Demographic rates were calculated from individual animal histories compiled by the Center for Whale Research^57^, using data collected from 1976 through 2014. The time series begins when the population was depleted by live-captures for display in aquariums^60^. The time series therefore includes periods of moderate population growth (1976 to 1993), subsequent decline, and approximate stability. Demographic rates were estimated for the age-class groupings used in recent models^24,61^, except that we set an upper limit for female breeding at 45 y rather than 50 y, because no females in the population have been documented to produce calves at older ages. Thus, we calculated survival and (for adult females) fecundity rates for calves (first year), juveniles (defined as from 1 y through 9 y of age), young mature females (10–30 y), older reproductive females (31–45 y), post-reproductive females (46 y and older), young mature males (10–21 y), and older males (22 y and older). Killer whales can survive many years after reproductive senescence, but estimating maximum longevity is difficult in such a long-lived species^62^. We set an upper limit of age to 90 y in our models, although only about 2% of females would be expected to reach this age, and only about 2% of males (with higher mortality) would be expected to exceed 50 y. Females stop breeding long before the maximum age, so the long-term population growth would not be affected by the upper age limit unless post-reproductive females benefit the pod in ways other than through their own reproduction.

Mortality for each age-sex class was averaged across the 39 years of data to obtain mean annual rates. We did not try to partition observed mortality into presumed causes of death. The use of these historic data for our Baseline model makes the implicit assumption that the frequency of deaths due to the various causes remains the same as has been observed across recent decades. The variation in mortality observed across years has two components: 1) environmental variation (fluctuations in the probability of survival), and 2) demographic stochasticity (binomial variation in individual fates). To determine how much of the observed variation was due to environmental variation, the variance due to demographic stochasticity can be calculated from the expectation for a binomial process, and then subtracted from the total variation across years. Calculated annual mortality rates (and environmental variation) ranged from a low of 0.97% (SD = 0) for young adult females to 17.48% (SD = 17.96) for calves. Although the lack of evidence for annual variation in the mortality adult females beyond that expected from random sampling of a constant probability might seem optimistic, for long-lived species a low level of annual variation in rates would have negligible effect on long-term population trajectories. We confirmed through sensitivity tests (Supplementary Information) that the environmental variation entered into the population model has no effect on our results.

The breeding system is polygamous, with some males able to obtain multiple mates, females mating with different males over their lifetimes, and mating between and within pods. Males become sexually mature (actively breeding, which may occur several years after they are physiologically capable of breeding) from 12 to 18 y of age. Thus, in the model, each male was assigned an age of sexual maturity by randomly selecting a value from 12 to 18. Variance in reproductive success among individual females and males will cause genetic diversity to be depleted faster and inbreeding to accumulate faster than would occur if mating was assumed random. Information is available on male mating success^51^, and we incorporated variation in male and female reproductive success in the model (Supplementary Information). Our models project an effective population size that is 37% of the total size, close to an estimate obtained from genetic data^58^.

Breeding rates, expressed as the proportion of the females of an age class that produce a calf each year, were calculated from annual census data. Rates ranged from 0% for post-reproductive females (age >45 y), to 7.88% (SD = 4.15) for older adult females (age 31–45 y), to 12.04% (SD = 3.54) for young adult females (age 10–30 y).

The upper limit on population size was set to 300, so that carrying capacity (K) would not restrict future population growth except under the best conditions tested. In the projections of current or expected conditions, the SRKW populations never reached this limiting size, and rarely exceeded 150 animals in any of the independent iterations of each simulation. Population recovery was assessed by the mean growth rate each year calculated before any carrying capacity truncation. Thus, the growth rate reflects the demographic potential and is not affected by the limit on population size in the model.

The SRKW population was projected for 100 years. For the initial exploration of parameter uncertainty, the simulation was repeated in 10,000 independent iterations to obtain high precision in mean and variance estimations. For comparisons among alternative management scenarios, less iteration is needed to obtain the relative influence of input values, and tests were run with 1,000 iterations. Sensitivity tests were conducted by varying each basic demographic rate (life table values for fecundity and mortality) over a range of ± 10% around the baseline value. For several model variables that describe other aspects of the population dynamics and are also very uncertain, a wider range of values was tested (see Supplementary Information).

### Individual Threats

We explored the effects of three threats identified in the recovery strategies. For each of prey abundance, noise disturbance, and PCB contaminants, we scaled impacts such that the estimated current level of the threat resulted in the mean demographic rates reported over recent decades. Effects of prey limitation were modelled using published relationships linking inter-annual variability in Chinook salmon to inter-annual variability in calf and adult mortality^63^ and fecundity^13,61^. A prey index was calculated by dividing the total salmon abundance in each year by its average abundance over the 1979–2008 period^63^. The relationship of mortality to prey abundance was modelled with a multiplier of baseline mortality that is a linear function scaled to 1 when salmon abundance was at the mean observed level over period of observation: MortalityFactor = 3.0412 – 2.0412 * PreyIndex. The relationship of birth rate to prey was modelled with logistic functions, with the intercept scaled to yield the observed birth rates for young females (12.04%) and older females (7.88%) when PreyIndex = 1. For relationships of form BirthRate = exp(A + B*PreyIndex)/[1 + exp(A + B*PreyIndex)], the function parameters were A = −3.0 and B = 1.0 for young females, and A = −3.46 and B = 1.0 for older females. (See Supplementary Information for more details on these relationships.) To explore the impacts of prey abundance across a range of plausible values, we varied the prey index from approximately the lowest level (0.60) reported since 1978 to approximately the highest level (1.30).

Effects of noise on demography were modelled using the approach outlined in previous analyses of loss of acoustic communication space^4,64^. We used summertime observations to estimate the proportion of time boats were present (during daylight hours) while the whales were foraging and the reduction in foraging expected with that amount of acoustic disturbance. We calibrated the model of noise impacts so that the mean Baseline demographic rates are obtained at the reported level of disturbance. We then simulated the relative change in foraging time and consequently demographic rates across the spectrum from no noise impact at all, to the upper limit expected if boat disturbance increased from current, already high, levels to 100% of time. We do not have data on the amount of acoustic disturbance in the winter feeding areas, but the modelling based on observed summertime disturbance provides a means to project a range of population consequences if changes in disturbance overall mirror those that are possible in the summertime habitat. Land-based observations have shown that SRKWs reduce their time spent feeding in the presence of boats by 25%^65^. Vessels are present 85% of the daytime, and SRKWs are foraging in the presence of vessels an estimated 78% of that time. Thus, for the 85% current (baseline) exposure to vessels, feeding is expected to be reduced by 16.6% ( = 85% × 78% × 25%) due to disturbance by boats. To translate the reduction in feeding into its demographic consequences, we multiplied the prey index by a factor of (1 − 0.195 * Noise)/(1 – 0.166) to obtain the proportional availability of prey. This proportion is thus 1 in the current, baseline conditions (Noise = 0.85), 0.965 when vessels are always present (Noise = 1.00), and 1.20 assuming no disturbance from vessels. The noise-modified index of prey availability was then used to determine the consequent mortality and fecundity rates. We recognize that anthropogenic noise can also have less direct effects on wildlife, including disruption of social behaviours and even impeding responsiveness to other sensory modalities^66^.

Our model of accumulation, depuration, and impact on calf survival of PCBs was based on the approach described by Hall *et al*.^67,68^ with modifications in rates for SRKW^69^. Calves obtain their initial load of contaminants from their dams through gestation and lactation, and females producing calves thereby depurate an estimated 77% of their contaminants^67^. Otherwise, males and non-breeding females accumulate PCBs in the blubber of at a rate that we varied from 0 to 5 ppm/y in our tests. Few data are available on PCBs in the SRKW population with which to calibrate the model of PCB bioaccumulation, and the levels of PCBs reported in SRKW might have been dropping slowly in recent years. Reported levels in adult female SRKW range from 55 ± 19 ppm sampled in 1993–1996, 37 ± 42 ppm sampled in 2004–2007, and 30 ± 31 ppm sampled in 2008–2013^30^. Our population model generates a mean 28, 55, and 81 ppm PCBs in adult females when bioaccumulation rate is 1, 2, and 3 ppm/y, respectively. Effects of maternal PCB load on calf mortality were modelled using a logistic response function (survival = exp(2.65 − 0.02 * PCB)/[1 + exp(2.65 − 0.02 * PCB)]), fitted to the two observed data points for SRKW (survival = 0.8252) and the nearby northern resident killer whales (survival = 0.9218)^24^, with the mean PCB levels (55.4 ppm and 9.3 ppm, respectively)^70^ reported from the time period in the middle of the span over which mortality rates were calculated. If we use the more recent, lower estimates of PCB loads in SRKW to estimate the impacts, our response function would have a steeper slope. There are not yet sufficient data on effects of PCBs on other demographic rates to allow inclusion of any other effects of PCBs (or other contaminants) in our PVA model.

### Cumulative Threats

We modelled two scenarios to represent the cumulative impacts of possible increases in threats, based in part on a recent environmental impact assessment submitted to Canada National Energy Board^45^ evaluating effects of a proposed oil pipeline and associated tanker traffic. For the purposes of this PVA, projected increases in anthropogenic threats are not meant to mimic any one industrial development, but rather a general process of industrialization reflecting the number of port expansions, pipeline proposals, and liquefied natural gas terminal proposals pending for the BC coast^4^. For a low level scenario, we used the catastrophe option in Vortex to add the possibility of large (>16,500 m^3^) and smaller (>8,250 m^3^) oil spills. The frequencies of a big spill (0.21% chance per year) and a smaller spill (1.08%) were based on an industry projection of the likelihood of such spills caused by proposed increase in tanker traffic^71^. Based on the percent overlap of oil coverage and critical habitat, we estimate that if a large oil spill were to occur, about 50% of the SRKWs would be killed due to direct exposure to the oil. We estimate that 12.5% of the SRKWs would be killed by exposure to oil from a smaller spill. For a scenario with higher level impacts of development, we doubled the frequency of oil spills.

These energy development scenarios also included an increase in vessel noise and disturbance of feeding, with the current vessel presence of 85% of time increased to 92.5% in the low level scenario and to 100% in the high level scenario. We also included a probability of additional deaths of killer whales due to ship strikes, with one death per decade in the low level and two deaths per decade in the high level scenario. Although some persistent organic pollutants might increase under increased industrial activity in the SRKW habitat, PCBs have been phased out of production and are in decline in at least some fish species in low-development basins^72^. Lacking data on likely long-term trends in the contaminant loads of SRKW prey, we did not include any change in such pollutants in these scenarios.

Climate change is projected to cause a decline in Chinook abundance^28^, and we modelled this possibility with a projected 25% (low scenario) or 50% (high scenario) decrease in Chinook over the next 100 years.

### Demographic Management and Threat Management scenarios

We used the PVA to simulate how much improvement in demographic parameters or how much reduction in anthropogenic threats, singly or in combination, would be required to reach a stated recovery objective of sustained annual population growth of 2.3% for 28 years^11^. In calculating the growth for these models, we started the tally 20 years into the simulation to avoid short-term demographic fluctuations as the age structure adjusts to new demographic rates, and growth was tallied over the subsequent 28 years. For the set of Demographic Management scenarios, we assessed the relationship between improved demography and population growth. Birth rate was incremented by 1.1x, 1.2x, 1.3x, 1.4x, and 1.5x, whereas calf mortality and adult mortality were decreased by 0.9x, 0.8x, 0.7x, 0.6x, and 0.5x. Next, in Threat Management scenarios, we modelled the effects of reduced threats, with the consequences resulting from the functional relationships to demography. We increased salmon abundance (up to the highest level of the Chinook index observed between 1979 and 2008, namely 1.3 times the long-term average). We simulated the improved demography if acoustic disturbance were reduced or eliminated. We considered the population consequences of improved calf survival resulting from reduction of PCBs, testing rates of future accumulation in SRKW from the estimated current 2 ppm/y to down to 0 ppm/y. Finally, we tested scenarios that both reduced acoustic disturbance by half and increased salmon abundance up to 1.3x.

## Electronic supplementary material

Supplementary Information
