# Supplementary material for: Evaluating anthropogenic threats to endangered killer whales to inform effective recovery plans
Source: Sci Rep. 2017 Oct 26;7:14119. doi: 10.1038/s41598-017-14471-0 (PMC5658391; doi:10.1038/s41598-017-14471-0)
Supplement: Supplementary file 1 — Supplementary Information [file 41598_2017_14471_MOESM1_ESM.doc]

# Supplementary Information -- Evaluating anthropogenic threats to endangered killer whales to inform effective recovery plans

Robert C. Lacy, Rob Williams, Erin Ashe, Kenneth C. Balcomb III, Lauren J. N. Brent, Christopher W. Clark, Darren P. Croft, Deborah A. Giles, Misty MacDuffee, Paul C. Paquet

**Expanded modelling methods**

**Primary data source for baseline demographic rates**

Demographic rates were calculated from the database on individual animal histories compiled by the Center for Whale Research^57^. Data from 1976 through 2014 were used. The SRKW population experienced moderate population growth from 1976 to 1993, and experienced a moderate decline subsequently. This may indicate that some aspects of the habitat declined in quality, or the change in trajectory may have been due to random fluctuations in reproduction and survival, rather than indicating any long-term trend. It might also be that the population peaked when it reached an ecological carrying capacity – the number of animals that can be supported by the available resource base. By including data back to 1976 for calculation of demographic rates, therefore, we may be providing an optimistic snapshot of current conditions. However, to restrict analyses to only the demography experienced in more recent years could under-estimate the capacity of the population to grow.

Vortex is an individual-based model, with the fate of each animal tracked through its lifetime. In contrast to population-based models or analytical calculations of average population growth, individual-based models can include individual variation in reproductive success, loss of genetic diversity, inbreeding, inbreeding avoidance, and any demographic consequences of these processes. We started the projections of population dynamics with the actual ages and sexes of the killer whales currently living in 2015. We also specified the mother of each animal, when it was known (for 76 of 80 living animals) ^51^. These designations of maternity are important, because we specified in the simulation model that females would not mate with their father, son, or maternal half-sibling. For the oldest animals in the population, we do not know mothers or other close relatives (other than their own offspring). Thus, the analyses will underestimate the accumulation of inbreeding, because living animals may be related in ways not documented.

Because the sample sizes are necessarily small, the data are not sufficient to determine accurate estimates of demographic rates for each annual age class. We therefore used the same age class groupings as were used in recent models^24,61^, except that we set an upper limit for female breeding at 45y rather than 50y. Not all SRKW calves and juveniles have been sexed, and the data do not indicate a significant difference in survival between males and females in these younger age classes, so survival was calculated with both sexes combined for calves and for juveniles. Survival rates of young adult females and young adult males have been better than older females and males, so it is appropriate to consider these age classes separately in the analyses. Killer whales can survive many years after reproduce senescence^62^. Although it is not known what age they can reach, and estimates of birth dates for the older animals in the population are uncertain, one female has been estimated to be 104 years old. The next oldest females are estimated to be 87y and 50y. We set an upper limit of age to 90y in our models. Although age estimates for the oldest animals are highly uncertain, the upper limit that we place on age in the model will have no impact on long-term population growth in our model. Post-reproductive females might provide benefits to the pod other than through reproduction, but we do not have quantitative data on such effects that would allow us to include them in the population model.

**Reproductive system**

The breeding system is polygamous, with some males able to obtain multiple mates, and females mating with different males over their lifetimes. There is evidence that there is variation among males and among females in reproductive success. Genetic evidence indicates that the effective population size is about 1/3 of the total size^58^, as would occur if some animals are more successful breeders than others. To represent this phenomenon in the PVA model, we assigned each male a score from 0 to 1 as his likelihood of being a capable breeder (i.e., with full access to breeding females) each year. The mean was set at 0.50, with a SD of 0.20 in a beta-distributed distribution. This leads to about 75% of offspring being sired by 15% of the males in our model. Similarly, we assigned an individual reproductive success score to each female, used to represent the factor by which the female’s probability of breeding each year was below (score <1) or above (score >1) the overall mean for females of her age. These scores for variation in female reproductive success had mean = 1.0 and SD = 0.2, created by sampling from a beta distribution (mean = 0.50, SD = 0.1) and doubling those values. The beta distribution was chosen over a normal distribution to ensure that values were bounded between 0 and 1.

Genetic data indicate that females usually mate with males from the other pods, but occasionally do mate within their own pod^58^. However, no cases of father-daughter, mother-son, or maternal half-sibling matings were reported in that study, even though the within-pod matings would make such inbreeding statistically likely (given the small population size) if mating were random. To include this inbreeding avoidance in the model, we specified that females would not mate with their fathers, sons, or brothers or half-brothers from the same mother. Matings between paternal half-siblings and more distant relatives were allowed in the model. The avoidance of very close inbreeding will reduce the extent of damage caused by inbreeding in the closed SRKW population, but could also lead to a situation in which some females might be unable to find a suitable mate if the population becomes small enough so that no unrelated males are available.

**Reproductive rates**

Breeding rates – expressed as the proportion of the females of an age class that produce a calf each year – were calculated to be:

12.04% for young adult females (age 10-30), with SD = 3.54

7.88% for older females (age 31-45), with SD = 4.15

0% for post-reproductive females (age > 45)

The above standard deviations expressing variation in rates across years were adjusted by removing the part of the observed variation that would be expected to be due to the demographic stochasticity (chance fluctuations in numbers breeding even when probabilities of breeding are constant), following methods in Lacy et al.^19^. Our estimated fecundities are a little higher than those estimated from the 1987-2011 data^24^. Our estimates of annual variation are lower because we removed the demographic stochasticity.

**Mortality**

Mortality rates were calculated from the tallies of deaths each year from 1976 through 2014. The mortality rate for each age-sex class was averaged across the 39 years of data to obtain the mean annual rates. Calculated annual mortality rates and environmental variation (represented as a standard deviation) were as follows:

Calves: 17.48% (SD = 17.96)

Juveniles: 2.15% (SD = 2.42)

Young adult females: 0.97% (SD = 0; i.e., the variation in mortality across years was no greater than expected based on the demographic stochasticity if the probability of death is constant over time)

Older adult females: 2.25% (SD = 0)

Post-reproductive females: 6.29% (SD = 7.77)

Young adult males: 3.03% (SD = 2.53)

Older adult males: 9.80% (SD = 8.68)

The above mortality rates are all a little better (i.e., lower) than previously published rates^24^, which is due to their use of data from 1987-2011, which omits a prior decade that had better demographic rates. The estimates of annual variation above are all considerably lower than previous estimates^24^ because of our removal of the expected demographic stochasticity from the annual variation in observed death rates.

**Impacts of inbreeding**

Long-term pedigree information are not available from which the effect of inbreeding could be determined for killer whales or for any cetacean species. The reported avoidance of mating between some closely related pod members, however, indicates that the species has reduced fitness when inbred. Lacking data on the severity of inbreeding depression in cetaceans, we assumed in the model that the effect of inbreeding on reproductive success (birth rate and calf survival) is the same as the mean value reported (6.29 “lethal equivalents”) in a survey of impacts on wild species^59^. Inbreeding might also affect other components of fitness – such as longevity – but data on such effects are too few to allow estimation of typical impacts, and we therefore did not include those effects in our model.

We used the option in Vortex to model inbreeding depression as being caused by the presence of recessive lethal alleles. Each initial animal in the model was assigned an average of 6.29 unique lethal alleles across its genome, distributed among founders according to a Poisson distribution. Vortex simulates the transmission of alleles at multiple loci through the pedigree, with offspring receiving an allele from each parent at each locus. Inbreeding depression is modeled by the death of any offspring that are homozygous for one of the recessive lethal alleles. See the Vortex manual^19^ for further description of how genetics and inbreeding depression are modelled.

**Sensitivity tests**

To determine the sensitivity of the model projections to the uncertainty in 11 demographic input variables, we ran 100 iterations each of 1000 combinations of input parameters sampled from ranges. The sampled parameters were evenly spaced across the range set for each, according to a “Latin hyperspace” design. This method of sampling provides high statistical power for determination of the effect of each parameter^26^. The ranges sampled for each variable were:

Inbreeding effects: Lethal equivalents = 3.145 to 9.435 (+ 50% around the baseline value)

Male variance in Reproductive Success: SD = 0 to 0.4

Female variance in Reproductive Success: SD = 0 to 0.4

Birth rate: + 10% around the baseline values

Annual variation in birth rate: + 10% around the baseline values

Calf mortality rate: + 10% around the baseline values

Annual variation in calf mortality: + 10% around the baseline values

Juvenile mortality rate: + 10% around the baseline values

Annual variation in juvenile mortality: + 10% around the baseline values

Adult mortality rate: + 10% around the baseline values

Annual variation in adult mortality: + 10% around the baseline values

The above ranges for sensitivity tests are different from a common practice of examining the amount of change in the population growth for a constant proportional change in each variable. However, the apparent standardization of sensitivities of growth to changes in variables is arbitrary. It is arbitrary because the same proportional change in different demographic rates might not be likely or achievable for the population of interest. For other model variables, such as the inbreeding depression, it is not clear that a 10% change in the value has the same meaning as a 10% shift in other demographic rates measured on very different and not always linear scales. Moreover, different results can be obtained if ages are binned into more or fewer age class categories for the calculations. Although strict comparison of the effect of proportional changes in model parameter values is often not meaningful, the sensitivity tests can be useful guides as to which model parameters are the most important determinants of population trajectories, over the range of values that are chosen for testing. Thus, it is often useful to vary parameter values by amounts that reflect our uncertainty in each input value.

To test the effect of inbreeding avoidance on population growth, we compared the baseline model to one in which there was no avoidance of close inbreeding.

**Modelling of impacts of Chinook salmon abundance**

A large body of evidence has shown a strong relationship between abundance of Chinook salmon and demographic rates for killer whales^13,14,24^. In the previous studies, details of the data used to quantify Chinook abundance, the co-factors considered, and the statistical methodologies differ in some key respects. One study examined fecundity for young and old females separately and examined impacts on survival rates of different age classes^24^, whereas others^13,14^ assessed relationships with pooled demographic data. Each derived an index of relative prey abundance, but the salmon stocks included and the period over which the indices were averaged differed among studies. Studies have examined the relationships with various time lags (0 to 2 years), although for projecting long-term population viability in simulation models, it is not important if changes in fecundity and survival due to changing Chinook abundance occur in the year of the Chinook index or with a lag of a year or two.

All three studies above identified large effects of Chinook salmon abundance on SRKW demographic rates. Two regression models are available that link the relationship between salmon abundance and logit(birth rate)^13,24^. We rescaled the regressions provided by both studies so that Chinook abundance index was set to 1.0 for the average over the span of data (1976-2014 for our demographic analyses), and so that the regression predicts the observed long-term average birth rate when Chinook index = 1. After that rescaling, the relationship for overall fecundity and for fecundity of the younger (more productive) females are very similar to the previous studies^13,24^ and are both approximately:

logit(birth rate) = -3.0 + Chinook (1)

in which Chinook is the abundance relative to the long-term average.

This relationship leads to a predicted shift from a birth rate of 12% to 18.5% if the Chinook abundance increases by 50%, and a shift down to 7.6% if prey abundance decreases by 50%. A linear relationship derived by Ford et al.^14^ would lead to smaller but still significant shifts (up to 13.3% and down to 9.2%, respectively) in the birth rate.

There is evidence for a stronger effect on fecundity of older females in relation to the Puget Sound terminal run, but a similar slope for Fraser + Puget abundance^24^. If the intercept in equation (1) is adjusted so that the fecundity of the older breeding females is correctly predicted at the baseline of Chinook = 1, then the relationship for that age class becomes approximately:

logit(birth rate) = -3.46 + Chinook (2)

Ford et al.^14^ provide the relationship of mortality to Chinook abundance in a form that is easily transferred to the Vortex model, namely:

Mortality index (relative to the expected value) = 4.0066 - 2.6504 x Chinook (relative abundance index) (3)

Rescaling this relationship so that the mortality index is 1 when Chinook = 1 gives:

Mortality index (relative to the baseline) = 3.6504 - 2.6504 x Chinook (4)

This relationship translates to a 26% increase in mortality for each 10% decrease in Chinook abundance. We used equation (4) to relate Chinook salmon abundance to SRKW mortality in our PVA.

The rescaling of the relationships above was confirmed to produce the same population growth in the Vortex model (r = -0.002, with SD = 0.045) as was projected from the Baseline model. These general relationships can then be used for projecting the primary impacts of changing Chinook abundance on population viability. We note that other regressions might be more appropriate for identifying the consequences to SRKWs of losing individual salmon stocks (“runs”), or the benefit of restoring runs in particular streams. The above-mentioned equations are appropriate for looking at broad-scale changes in SRKW habitat.

**Sensitivity testing of model parameters**

We ran simulations with higher and lower values of each demographic parameter to determine the importance of each to population growth. For each model parameter that was tested, Table S1 gives the value used in the baseline model (birth rate calculated separately for younger and older females), the minimum and maximum values of the range that was tested, the population growth rates that resulted from the min value (r-min) and max vale (r-max) for that parameter while all other parameters were held at their baseline, and the % of variance in population growth (as a percent of the total variation accounted for by the varied parameters) due to variation in that parameter when all other parameters were simultaneously and independently varied across their ranges of uncertainty.

Table S1. Values for each parameter in the sensitivity analysis, their effect on projected population growth, and proportion of variation explained by variation in that parameter. The residual variance is that due to non-linearities of and interactions among effects.

| Parameter | Baseline | Min tested | Max tested | r-min | r-max | %variance |
| --- | --- | --- | --- | --- | --- | --- |
| Inbreeding depression | 6.29 | 3.145 | 9.435 | -0.0005 | -0.0029 | 6.1 |
| Variance in male RS | 0.20 | 0.00 | 0.40 | -0.0017 | -0.0023 | 0.4 |
| Variance in female RS | 0.20 | 0.00 | 0.40 | -0.0017 | -0.0019 | 0.0 |
| Birth rate (young;old) | 0.1204  0.0788 | 0.1084  0.0709 | 0.1324  0.0867 | -0.0062 | 0.0019 | 77.2 |
| SD in birth rate | 0.0354  0.0415 | 0.0319  0.0374 | 0.0389  0.0457 | -0.0020 | -0.0018 | 0.0 |
| Calf mortality | 0.1748 | 0.1573 | 0.1923 | -0.0012 | -0.0029 | 3.2 |
| SD in calf mortality | 0.1796 | 0.1616 | 0.1976 | -0.0017 | -0.0019 | 0.0 |
| Juvenile mortality | 0.0215 | 0.0194 | 0.0237 | -0.0011 | -0.0028 | 3.1 |
| SD in juv. Mortality | 0.0242 | 0.0218 | 0.0266 | -0.0015 | -0.0019 | 0.0 |
| Adult mortality | various (see text) | base * 0.9 | base * 1.1 | -0.0010 | -0.0028 | 6.4 |
| SD in adult mortality | various (see text) | base * 0.9 | base * 1.1 | -0.0018 | -0.0018 | 0.0 |
| Residual |  |  |  |  |  | 3.5 |

When inbreeding avoidance was dropped from the Baseline model, mean population growth was reduced very slightly, from r = -0.0020 to r = -0.0024.

**References**

All references cited in this Supplementary Information are given in the main manuscript.
